# Supplementary material for: Interpersonal Communication in Intensive Care Units: A Qualitative Study on Family Members’ Experiences in a Turkish Public Hospital
Source: Healthcare (Basel). 2025 Nov 28;13(23):3100. doi: 10.3390/healthcare13233100 (PMC12692004; doi:10.3390/healthcare13233100)
Supplement: Supplementary file 1 [file healthcare-13-03100-s001.zip › healthcare-3929290-supplementary.pdf]

Supplementary File S1: COREQ checklist

Item No	Guide Questions/Description	Reported on Page
Domain 1: Research team and reflexivity		
Personal Characteristics		
1. Interviewer/ facilitator	Which author/s conducted the interview or focus group?	Page5, Study Population
2. Credentials	What were the researcher's credentials? E.g., PhD, MD	Page5, Study Population
3. Occupation	What was their occupation at the time of the study?	Page5, Study Population
4. Gender	Was the researcher male or female?	Page5, Study Population
5. Experience and training	What experience or training did the researcher have?	Page5, Study Population
Relationship with participants		
6. Relationship established	Was a relationship established prior to study commencement?	No
7. Participant knowledge of the interviewer	What did the participants know about the researcher? e.g. personal goals, reasons for doing the research?	Page 6, Data Collection
8. Interviewer characteristics	What characteristics were reported about the interviewer/facilitator? e.g. Bias, assumptions, reasons and interests in the research topic	Supplementary Materials, Appendix 1
Domain 2: study design		
Theoretical framework		
9. Methodological orientation and Theory	What methodological orientation was stated to underpin the study? e.g. grounded theory, discourse analysis, ethnography, phenomenology, content analysis	Page 3, Study Design

Item No	Guide Questions/Description	Reported on Page
Participant selection		
10. Sampling	How were participants selected? e.g., purposive, convenience, consecutive, snowball	Page 4, Study Population
11. Method of approach	How were participants approached? e.g., face-to-face, telephone, mail, email	Page 4, Study Population
12. Sample size	How many participants were in the study?	Page 4, Study Population
13. Non-participation Setting	How many people refused to participate or dropped out? Reasons?	No
14. Setting of data collection	Where was the data collected? e.g., home, clinic, workplace	Page 5, Data Collection
15. Presence of nonparticipants	Was anyone else present besides the participants and researchers?	No
16. Description of sample	What are the important characteristics of the sample? e.g. demographic data, date	Page7 Data Collection
Data collection		
17. Interview guide	Were questions, prompts, and guides provided by the authors? Was it pilot tested?	Supplementary Materials
18. Repeat interviews	Were repeat interviews carried out? If yes, how many?	Page 5, Data Collection
19. Audio/visual recording	Did the research use audio or visual recording to collect the data?	Page 6, Data Collection
20. Field notes	Were field notes made during and/or after the interview or focus group?	Page 6, Data Collection
21. Duration	What was the duration of the interviews or focus group?	Supplementary Materials
22. Data saturation	Was data saturation discussed?	Page 6, Data Collection

Item No	Guide Questions/Description	Reported on Page
23. Transcripts returned	Were transcripts returned to participants for comment and/or correction?	No
Domain 3: analysis and findings		
Data analysis		
24. Number of data coders	How many data coders coded the data?	Page 5, Study Population
25. Description of the coding tree	Did the authors provide a description of the coding tree?	No
26. Derivation of themes	Were themes identified in advance or derived from the data?	Page 6, Thematic Analysis
27. Software	What software, if applicable, was used to manage the data?	Page 6, Data Collection
28. Participant checking	Did participants provide feedback on the findings?	No
Reporting		
29. Quotations presented	Were participant quotations presented to illustrate the themes/findings? Was each quotation identified? e.g., participant number	Page 11, Table 6; Page 12, Table 7
30. Data and findings consistent	Was there consistency between the data presented and the findings?	Page 14, Discussion
31. Clarity of major themes	Were major themes clearly presented in the findings?	Page 7, Table 2.
32. Clarity of minor themes	Is there a description of diverse cases or a discussion of minor themes?	Page 8, Table3; Page 9, table 4; Page 9-10, Table 5; Page 11, Table 6; Page 12, Table 7.
